# RTK: efficient rarefaction analysis of large datasets

**DOI:** 10.1093/bioinformatics/btx206

**Published:** 2017-04-07

**Authors:** Paul Saary, Kristoffer Forslund, Peer Bork, Falk Hildebrand

**Affiliations:** 1 Structural & Computational Biology Unit, EMBL, Heidelberg, Germany; 2 MMPU, University of Heidelberg and European Molecular Biology Laboratory, Heidelberg, Germany; 3 Max Delbrück Centre for Molecular Medicine, Berlin, Germany; 4 Department of Bioinformatics, University of Würzburg, Würzburg, Germany

## Abstract

**Motivation:**

The rapidly expanding microbiomics field is generating increasingly larger datasets, characterizing the microbiota in diverse environments. Although classical numerical ecology methods provide a robust statistical framework for their analysis, software currently available is inadequate for large datasets and some computationally intensive tasks, like rarefaction and associated analysis.

**Results:**

Here we present a software package for rarefaction analysis of large count matrices, as well as estimation and visualization of diversity, richness and evenness. Our software is designed for ease of use, operating at least 7x faster than existing solutions, despite requiring 10x less memory.

**Availability and Implementation:**

C ++ and R source code (GPL v.2) as well as binaries are available from https://github.com/hildebra/Rarefaction and from CRAN (https://cran.r-project.org/).

**Supplementary information:**

Supplementary data are available at *Bioinformatics* online.

## 1 Introduction

A common task in ecology and microbiomic data analysis is to count and compare the occurrences of different organisms throughout different samples, resulting in taxa count matrices. Accounting for biases due to uneven depth of sampling between sites or time points is a major analytical challenge. Rarefaction is a data normalization technique designed to cope with such unequal sampling efforts, by subsampling to the same rarefaction depth for all samples, thus simulating equal sampling effort. This allows calculation of comparable diversity estimators and enables collectors curves, to estimate total expected diversity.

Although several rarefaction implementations in microbiomics exist (e.g. vegan (Oksanen *et al.*, 2016), QIIME (Caporaso *et al.*, 2010), mothur (Schloss *et al.*, 2009)), these often work poorly for very large datasets because of memory requirements, processing limitations and program design (see Supplementary Material), which requires custom parsing scripts and the use of special hardware to do rarefactions. Here, we present the rarefaction toolkit (RTK), which can perform fast rarefaction on very large datasets comprising millions of features even on a laptop computer, computes estimates of ecological diversity and provides appropriate visualizations of the results.

## 2 Implementation

RTK is implemented in C ++11 with an optional R interface, having two principal run modes: ‘memory’ and ‘swap’, the latter using temporary files to reduce memory footprint. Using asynchronous thread management, RTK can make use of modern multi-core processors. The algorithm works by transforming input counts into a vector of feature occurrences and shuffles it using the Mersenne Twister (Matsumoto and Nishimura, 1998) random number generator. A subset of this shuffled vector of length equal to the desired rarefaction depth is used to construct the rarefied sample and to estimate diversity. Multiple rarefactions are calculated, by reusing unused parts of the shuffled vector, guaranteeing unique sampling without wasting computational resources. From the rarefied matrix evenness, three diversity and five richness estimators are computed (see Supplementary Text). The R-package ‘RTK’ provides an interface and visualizations to the C ++ RTK, using the Rcpp package (Eddelbuettel and François, 2011).

## 3 Comparison to existing software

We used three tests to compare performance and memory consumption of RTK to vegan 2.4, mothur 1.38.1 and QIIME 1.9.1 on a Linux cluster with 1 TB RAM, using a single core. Other rarefaction programs were considered, but were not suited for high-throughput analysis (see Supplementary Material).

**Fig. 1:**
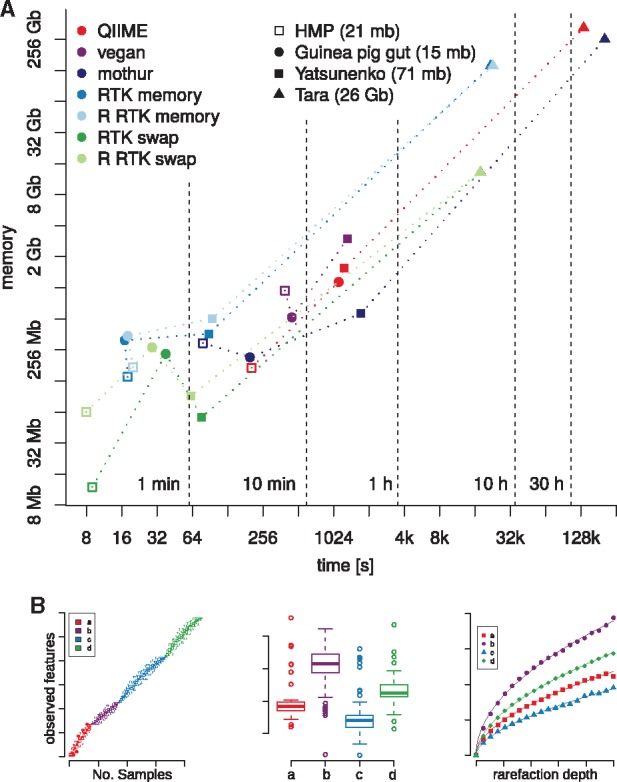
(**A**) Speed and memory requirements of different rarefaction programs. Four datasets were 20 times rarefied at 95% lowest sample count. Time and memory consumption of our implementation is consistently below that observed using mothur, vegan or QIIME for the same purpose. vegan failed processing the Tara table (see Supplementary material). (**B**) Plotting of collector curves as well as of rarefaction curves is implemented in the R-package (Color version of this figure is available at Bioinformatics online.)

Four published metagenomic datasets of different size were used: Two were human gut 16S OTU count tables termed Yatsuneko (Yatsunenko *et al.*, 2012) and HMP (Huttenhower *et al.*, 2012), both processed with the LotuS pipeline (Hildebrand *et al.*, 2014). We also reanalyzed two metagenomic datasets, termed Guinea pig gut (Hildebrand *et al.*, 2012) and Tara from *Tara* Oceans (Sunagawa *et al.*, 2015), using publicly available gene count matrices (see Supplementary Table S1 for statistics). We first computed the mean ecosystem richness over 20 rarefactions. For all dataset sizes RTK outperformed the other programs with regards to speed and memory requirement (Fig. 1, Supplementary Table S2). To rarefy the Tara gene matrix, all other programs required prohibitively large amounts of memory (>256 GB), while RTK required only a fraction of this (<10 GB), providing also a 5-fold increase in speed (Table 1, Fig. 1). Second, we tested performance when the number of repeated rarefactions to the same depth varied (Supplementary Fig. S2). vegan, mothur and QIIME had a linear increase in runtime with increasing repeats, whereas RTK runtime remained almost constant. Last, we tested multicore performance (only available in RTK), which reduced RTK runtime by a factor of three using 8 cores (see Supplementary Fig. S3).

**Table 1.** Time and memory consumption when rarefying the Tara gene abundance matrix five times to 2.3 M counts per sample, from 139 M counts on average per sample

Software (mode)	Runtime	Max. memory	Success
RTK (memory)	3:50 h	140 Gb	successful
RTK (swap)	3:30 h	8.5 Gb	successful
R RTK (memory)	3:30 h	140 Gb	successful
R RTK (swap)	3:05 h	8.7 Gb	successful
QIIME	21:50 h	339 Gb	successful
vegan	–	387 Gb	failed
mothur	17:30 h	262 Gb	successful

*Note:* While RTK could return the rarefied data, mothur only reports diversity.

## 4 Discussion

Rarefaction is a standard data normalization technique in numerical ecology, also useful to avoid false positive detection of rare features when comparing unequally sampled data (Supplementary Fig. S4, Supplementary Text). Rapid expansion in the size of microbiomic datasets makes rarefaction difficult to employ, due to speed and memory limitations. Here we present a software solution that is well-suited for state of the art microbiomics applications. It provides diversity estimators, various visualizations and statistics related to these, is easy and free to use, and scales better than presently available tools.

## Supplementary Material

Supplementary Data
