# Supplementary material for: RTK: efficient rarefaction analysis of large datasets
Source: Bioinformatics. 2017 Apr 7;33(16):2594–5. doi: 10.1093/bioinformatics/btx206 (PMC5870771; doi:10.1093/bioinformatics/btx206)
Supplement: Supplementary Data [file 20170327_rtk_supplement_btx206.pdf]

RTK - supplementary material

Paul Saary [1], Kristoffer Forslund [1], Peer Bork [1,2,3,4] and Falk Hildebrand [1]

March 27, 2017

[1]Structural & Computational Biology Unit, European Molecular Biology Laboratory (EMBL), Meyerhofstrasse 1, Heidelberg 69117, Germany. and
[2]Molecular Medicine Partnership Unit (MMPU), University of Heidelberg and European Molecular Biology Laboratory, Heidelberg, Germany.
[3]Max Delbrück Centre for Molecular Medicine, D-13125 Berlin, Germany.
[4]Department of Bioinformatics, University of Würzburg, D-97074 Würzburg, Germany.

Contents

1 Introduction **1**

2 Implementation and code **2**
2.1 Diversity estimates 2

3 Benchmarks **2**
3.1 Tara Ocean Project 2
3.2 Multi threading using the async function of C++11 5
3.3 Rarefaction of large datasets on consumer hardware 5
3.4 Using rarefaction to prevent false positive results 5

4 Extended methods **5**
4.1 Measuring memory consumption and speed 5
4.2 Comparing RTK to other solutions 6

5 Plot functions **8**

1 Introduction

When faced with unequal sampling efforts, the comparison of samples is dependent on proper sample normalization. For a number of applications, rarefaction or subsampling of short-read sequencing data is necessary for comparative analysis to be fair and robust. While others have argued against its routine practice on grounds that already scarce data should not be reduced further (McMurdie and Holmes, 2014), the practice is widely used, often drawing on routines in scripting languages for statistical analysis such as the vegan (Oksanen et al., 2016) package for R.

Feature count matrices are a basic data format used in numerical ecology. Also metagenomic and -transcriptomic analysis produces count data, wherein a large number of data points, such as short sequencing reads, are determined and assigned to categories for quantification, e.g. OTUs (operational taxonomic units) or a reference gene catalog. These categories can in turn be ordered into hierarchies corresponding to biological readouts of interest, such as the number of reads mapping to a particular broadly or narrowly defined bacterial taxon, or to a gene functional module. In many cases, analysis of an environmental or clinical dataset will then be proceeded by sta-

tistical tests for whether or not a particular set of categories are relatively enriched.

Variation in size between samples from multiple sequencing runs or different studies can be reduced by using rarefaction as implemented by us in the rarefaction toolkit (RTK). Such reduced variation in overall sample count is crucial for assessing comparable sample diversity, in the form of different measures of spread or entropy remaining after rarefying to a common overall depth.

2 Implementation and code

The RTK project is hosted on GitHub[1]. Binaries are provided under releases[2]. Bugs and feature requests can be reported directly on GitHub.

2.1 Diversity estimates

RTK provides the ability to compute several common diversity measures including richness, Pielou's evenness (Mulder et al., 2004), chao 1 (Chao, 1984), and the Shannon (Shannon, 1948), Simpson and inverse Simpson diversity (Simpson, 1949). The formulas and function of which are briefly presented here:

Richness reports simply the number of different species in a single sample by evaluating if a species is present or not. Applied to a gene count matrix this yields a gene richness estimate, as was recently linked in the case of gut microbial ecosystems to the BMI of the human host (Huttenhower et al., 2012).

Claude Shannon introduced the Shannon Diversity to quantify the entropy of a sample (Equation 1). The same measurement is known as the Shannon Entropy in information theory.

$$H' = -\sum_i p_i \cdot \ln pi_i \tag{1}$$

Where p_i is the probability of species n_i to occur in the sample space N ($p_i = n_i/N$).

Pielou's evenness describes the evenness across species in absolute numbers. Mathematically it can be described as

$$E = \frac{H'}{H'_{max}} \tag{2}$$

Where the evenness E is defined as the fraction of the Shannon diversity divided by the maximal possible Shannon diversity.

To asses the probability that two specimen picked at random are from the same type the Simpson index in the form of $\lambda = \sum_i p_i^2$ was suggested by Edward Simpson (Simpson, 1949). The inverse Simpson index is returned for each sample as $\frac{1}{\lambda}$.

To indicate if rare species (singletons F_1) are still expected to be discovered the chao 1 estimator can be used (Robert K Colwell and Coddington, 1994). It reports the fraction of singletons (species occurring exactly once) to doublets (species that are reported twice (F_2)) multiplied with the number of species present in the sample (S_{obs}) (Equation 3).

$$S_1 = S_{obs} \cdot \frac{F_1^2}{2 \cdot F_2} \tag{3}$$

The Incidence Coverage-based Estimator (ICE) and Abundance Coverage-based Estimator (ACE) were implemented after the fossil package (Vavrek, 2011) and yielded comparable results.

3 Benchmarks

3.1 Tara Ocean Project

The Tara Ocean project matrix used as an example has more than $40 \cdot 10^6$ OTU's and 243 samples (Table 1). Opening the table in R for vegan to process was successful, but vegan was then not able to process such large vectors and failed with this error message after already three hours of computation time and using over 380 Gb of memory (while 500 Gb of RAM was scheduled and available for this process):

```
  This is vegan 2.4-0
Error in all.equal.numeric(x,
round(x)) :
long vectors not supported yet:
eval.c:3047
Calls:  <Anonymous> -> identical ->
```

[1] https://github.com/hildebra/Rarefaction

[2] https://github.com/hildebra/Rarefaction/releases

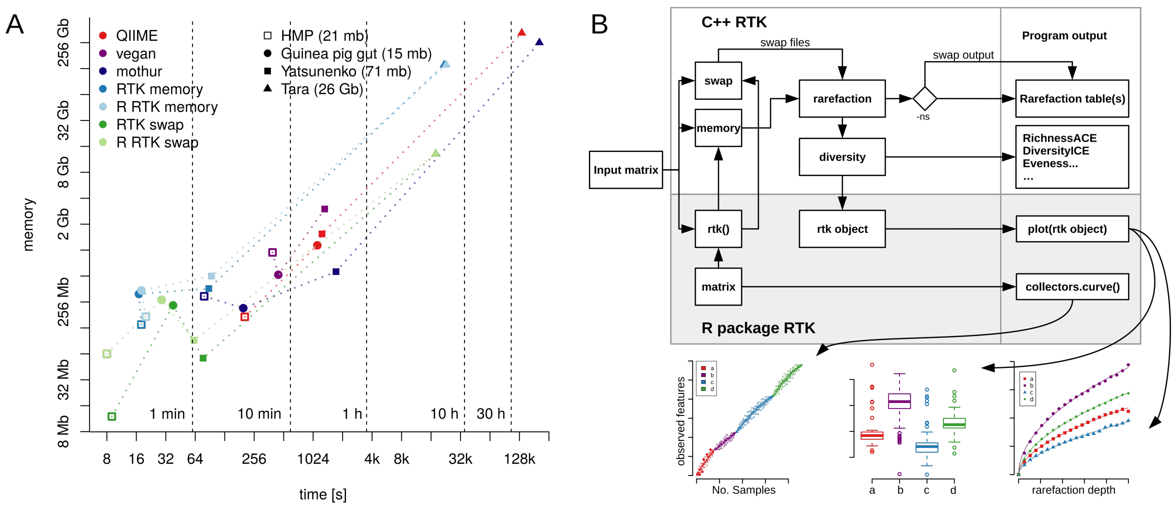

Suppl. Figure 1: (A) Speed and memory requirements of different rarefaction programs. Using input matrices of increasing file size, we measured the rarefied richness 20 times per sample at 95% of the lowest sample rarefaction depth. Time and memory consumption of our implementation is consistently below that observed using mothur, vegan or QIIME for the same purpose. vegan failed processing the Tara table even though enough computing resources were available (see Suppl.). (B) Implementation of the RTK package. RTK can be used either as a standalone C++ application or as an integrated R package. In either implementation two run modes, swap and memory, are available for generating either diversity measures, rarefied matrices or both. Plotting routines commonly used for diversity measurements are also implemented in our R-package.

Suppl. Table 1: The datasets used in this work to asses the performance of the presented RTK software solution for rarefaction of large datasets are all of different size and previously published.

abbreviation	file size	No. of Samples	No. of Features	citation
HMP	21 Mb	4798	2201	Huttenhower et al., 2012
Guinea pig gut	15 Mb	8	610834	Hildebrand et al., 2012
Yatsunenko	71 Mb	527	68311	Yatsunenko et al., 2012
Tara	26 Gb	243	40154823	Sunagawa et al., 2015

Suppl. Table 2: Raw data used to create Figure 1 in the corresponding application note. See Table 1 for more information on each dataset used.

dataset	file size	software	time [sec]	max. memory [Mb]
HMP	21 mb	mothur	79	298
Yatsunenko	71 mb	mothur	1753	576
Guinea pig gut	15 mb	mothur	198	217
Tara	26 Gb	mothur	209484	262449
HMP	21 mb	QIIME	205	172
Yatsunenko	71 mb	QIIME	1261	1580
Guinea pig gut	15 mb	QIIME	1130	1164
Tara	26 Gb	QIIME	138510	339000
HMP	21 mb	Vegan	394	963
Yatsunenko	71 mb	Vegan	1344	3075
Guinea pig gut	15 mb	Vegan	451	530
Tara	26 Gb	Vegan	-	-
HMP	21 mb	RTK (memory)	18	140
Yatsunenko	71 mb	RTK (memory)	88	366
Guinea pig gut	15 mb	RTK (memory)	17	317
Tara	26 Gb	RTK (memory)	22289	144809
HMP	21 mb	R RTK (memory)	20	173
Yatsunenko	71 mb	R RTK (memory)	94	513
Guinea pig gut	15 mb	R RTK (memory)	18	349
Tara	26 Gb	R RTK (memory)	23394	144890
HMP	21 mb	RTK (swap)	9	12
Yatsunenko	71 mb	RTK (swap)	77	57
Guinea pig gut	15 mb	RTK (swap)	38	234
Tara	26 Gb	RTK (swap)	18345	13386
HMP	21 mb	R RTK (swap)	8	64
Yatsunenko	71 mb	R RTK (swap)	62	92
Guinea pig gut	15 mb	R RTK (swap)	29	271
Tara	26 Gb	R RTK (swap)	18200	13467

```
all.equal -> all.equal.numeric
Execution halted
```

mothur, QIIME and RTK were able to open and process the large table and are thus represented in the main figure of the application note.

3.2 Multi threading using the async function of C++11

To use multiple threads and thus take advantage of new processor designs and the availability of multiple cores, nowadays present in normal laptops, desktop PC's and even cell phones, special software design is needed.

Asynchronous thread launching is implemented as the function `async` in C++11[3]. We make use of this function by allowing rarefaction and diversity measurement computation of multiple samples at once, launching a separate thread for each sample. This allows reduction of the computational time significantly after the file is loaded into the software (Figure 3).

For multi thread support RTK should be compiled with the `pthread` flag of the compiler (as defined by default in the makefile).

3.3 Rarefaction of large datasets on consumer hardware

Large datasets are always a challenge to process on consumer hardware as CPU, local storage space and memory is limited. RTK is able to rarefy the Tara matrix, which needs over 250 Gb of RAM when processed with mothur, QIIME or vegan, on a common Mac Book (version mid 2014) with a 2.8 Ghz intel Core i5 CPU, 16 Gb of RAM and an internal 500 Gb SSD.

The diversity measures for the 26 Gb large file were calculated in 7.5 h of computation time using only 13 Gb of memory and swap. Also five rarefied matrices were returned and written to the hard disk, each 20 Gb in size. Without writing rarefied tables to disk, rarefaction measures could be calculated in only 2 h using the same amount of memory.

[3] http://en.cppreference.com/w/cpp/thread/async

3.4 Using rarefaction to prevent false positive results

Based on how metagenomics techniques have a detection threshold for low-abundance organisms, we would expect comparative analysis of samples of different size to sometimes yield false but apparently significant differences between datasets with respect to abundance of such taxa. To benchmark the potential scope, we took an existing deeply sequenced dataset (the Yatsunenko dataset) and created a corresponding artificial dataset which is biologically identical but sequenced at lower depth by rarefying it (using vegan) to a tenfold lower total number of reads. Any significant differences in bacterial taxon abundance between these datasets, then, must follow solely from artifacts of the differences in read depth. We compared the abundance of each taxon in the samples in both datasets (original and rarefied), plotting the FDR-adjusted *Mann–Whitney U test* P-values of these tests in Figure 4, left item. As we see, there is a sizable number of features for which a rarefaction-naïve analysis falsely would report (and publish) an association. Rarefying all samples to the same size eliminates these artifacts, bringing the empirical and expected false discovery rate into agreement. We thus note that unless rarefaction is used, read depth differences such as exists between published metagenome datasets risks yielding sizable numbers of false positives, particularly in studies pooling samples from different sources for comparison or meta-analysis. An alternative approach for solving this normalization task would be to use proportionality-aware methods like edgeR (Robinson, McCarthy, and Smyth, 2010), thereby also avoiding loss of statistical power.

4 Extended methods

4.1 Measuring memory consumption and speed

To measure the runtime of a single experiment the execution time was estimated using the GNU time utility (version 1.7) provided by CentOS 6.5 with the parameter `-p` to re-

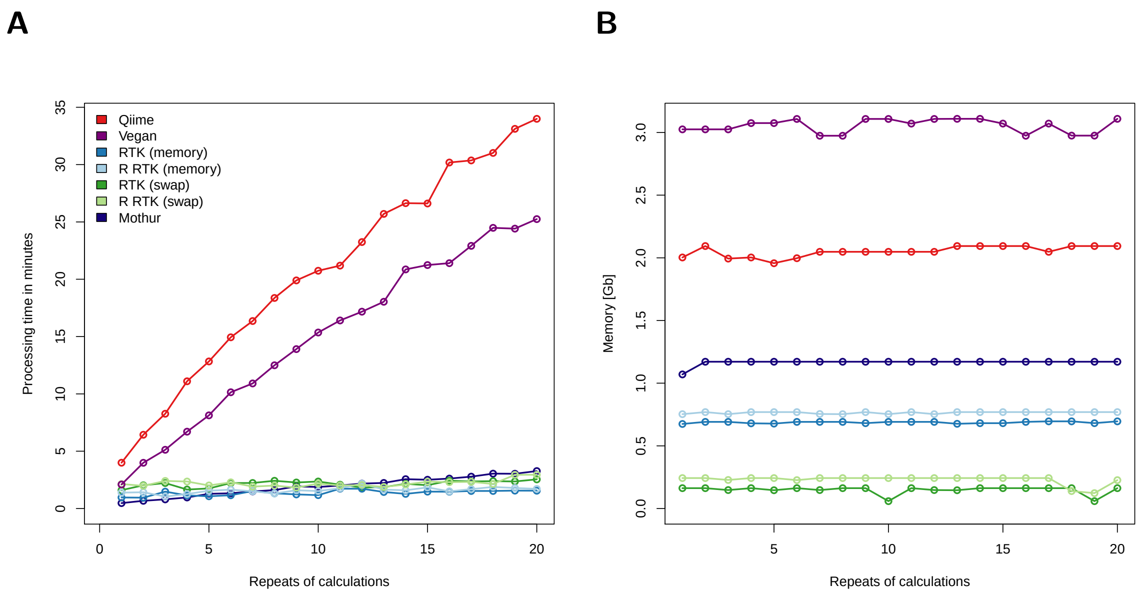

Suppl. Figure 2: To asses the performance of the presented package, the Yatsunenko (Yatsunenko et al., 2012) dataset was rarefied n times with different applications. The advantage of RTK becomes clear after a few repeats of rarefaction. While the memory footprint of any run mode is clearly below mothur, QIIME and vegan (see B), our solution has a constant runtime (compare A), nearly independent of the number of rarefactions, while mothur, QIIME and vegan show a steady increase in computational time needed.

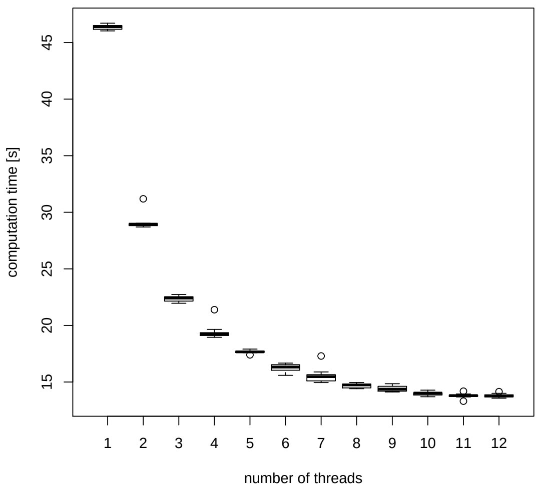

Suppl. Figure 3: Increasing the number of threads used can decrease the computational time needed drastically. In RTK multiple samples will be rarefied at once. In this example, using 8 threads reduced the computational time needed by a factor of three compared to using only a single thread. This effect might vary between datasets as parallelization is done on different samples after reading the file.

ceive the raw time in seconds.

The utility reports user, real as well as system time. To make comparisons possible across several experiments, if not specified differently, the time the software spend in user space was used across all measurements.

The maximal memory consumption was estimated by calling `ps -o vsz $pid` every second measuring the virtual memory size (vsz) of the process.

4.2 Comparing RTK to other solutions

As stated we compare RTK to multiple available rarefaction software tools. This would also include EstimateS (R. K. Colwell, 2013) as well as analytic rarefaction (Holland, 2003).

While evaluating EstimateS the software failed our requirements to process large files as it failed to even open the Tara matrix due to the available memory on the machine. As only a closed source GUI version for Mac OS and Windows is provided we were not able to run EstimateS on our server which would have provided an adequate environment to

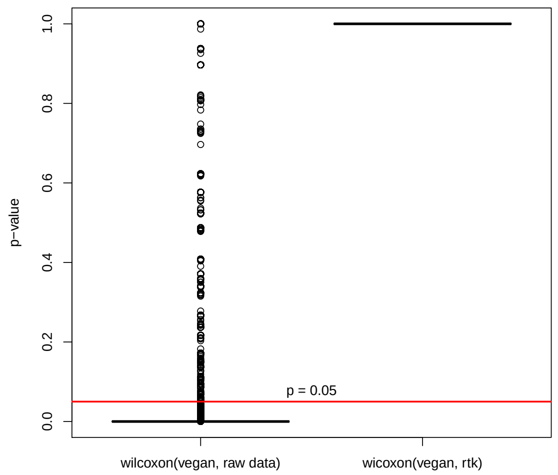

Suppl. Figure 4: Spurious conclusions of significant differences between datasets result from large read depth differences, and are eliminated through uniform rarefaction: To illustrate how read depth differences cause spurious false positive abundance differences, we created an artificial dataset from the Yatsunenko table, by reducing their effective read depth to 10% of the original number of reads per sample. The box plot shows FDR-adjusted *Mann–Whitney U test* Q-values from comparing the abundances of each taxon in the original dataset to the abundances of the same taxon in the depth-reduced, but otherwise identical, dataset. Since samples differ only in read depth, all apparent significances reflect artifacts. If no artifacts occur, we would expect no more than n% of FDR values to fall below any cutoff n%. The left side shows that under comparison of the original data set with its reduced counterpart, this expectation is violated: 86.4% of the features are instead found significantly different in abundance. On the right the same analysis was repeated following rarefaction of all samples to the same size. Here no such false significances are reported, highlighting the need for rarefaction when comparing samples of very different depth this way.

open larger files. Furthermore EstimateS 9 uses a different approach to rarefaction, and thus is not directly comparable to the presented solution, as classical rarefaction with defined sample sizes is not possible.

The same is in some way true for analytic rarefaction, as we were not able to open the described matrix with the software which is only provided as binaries for windows and via the App store of Apple for Mac OS.

Other available software for rarefaction, such as HPrare (Kalinowski, 2005) and ADZE (Szpiech, Jakobsson, and Rosenberg, 2008) focus on different file formats and use cases and thus they were not compared to the presented solution.

5 Plot functions

Rarefaction if often used as a tool to evaluate the sampling effort done. For that rarefaction and collector curves of the dataset are plotted.

RTK provides an easy to use R interface for such cases. After rarefaction to multiple depths it is possible to plot the rarefaction curve for all samples or groups of samples by simply calling `plot()` on the created object. This will result the in a plot as shown in figure 5.

For groups of samples the Kruskal–Wallis test is applied (Kruskal and Wallis, 1952), as shown above the plot, to assess significant differences in diveristy between given groups. Colors, symbols and fitting of the values can be specified as options which are documented in the package.

Grouping of the samples is done using the median between the grouped samples.

For fitting of the diversity measures three different fitting functions are provided: By default the *Arrhenius equation* (Equation 4) will be used, alternatively *Michaelis-Menten* (Equation 5) or the *logistic function* (Equation 6) can be applied.

$$k = A \cdot e^{\frac{E_a}{RT}} \tag{4}$$

$$v = \frac{V_{max}[S]}{K_M + [S]} \tag{5}$$

$$f(x) = \frac{L}{1 + \mathrm{e}^{-k(x - x_0)}} \tag{6}$$

Collector curves which display the increase in richness if randomly samples are added or removed to the dataset, can be plotted using a raw input matrix or an ,,RTK object”(Figure 5).

If rarefaction is only done for one depth a boxplot function for the resulting diversity measures at that single depth is also provided (Figure 6).

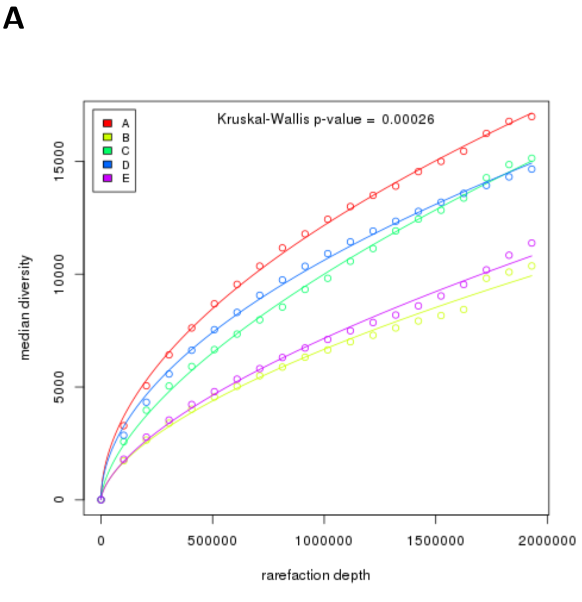

Suppl. Figure 5: The plotting interface allow the user to quickly asses the quality of the dataset. A) A rarefaction curve of the data can be performed directly on the rarefied dataset by calling the plot function on the returned object. Optional grouping, fitting and styling can be done directly in R. B) Collector curves can be plotted using rarefied or unrarefied datasets. The number of repeats can be specified to reduce computation time or increase accuracy.

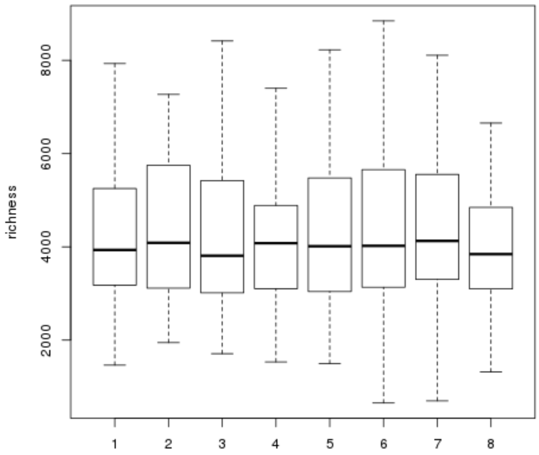

Suppl. Figure 6: Boxplots of (grouped) samples are created if rarefaction was done only for a single depth.

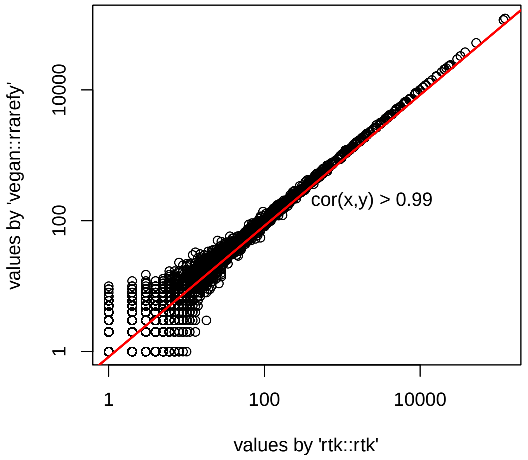

Suppl. Figure 7: Rarefaction of the same dataset with vegan and with RTK in R to the same depth results in a correlation of over .99, which proves the fitness of the rarefaction result presented. A linear fit between vegan and RTK values after rarefaction is drawn in red into the scatter plot.

References

Chao, Anne (1984). "Nonparametric estimation of the number of classes in a population". In: *Scandinavian Journal of statistics*, pp. 265–270.

Colwell, R. K. (2013). *EstimateS: Statistical estimation of species richness and shared species from samples. Version 9. User's Guide and application.* URL: http://purl.oclc.org/estimates.

Colwell, Robert K and Jonathan A Coddington (1994). "Estimating terrestrial biodiversity through extrapolation". In: *Philosophical Transactions of the Royal Society of London B: Biological Sciences* 345.1311, pp. 101–118.

Hildebrand, Falk et al. (2012). "A comparative analysis of the intestinal metagenomes present in guinea pigs (Cavia porcellus) and humans (Homo sapiens)". In: *BMC genomics* 13.1, p. 514.

Holland, Steven M (2003). "Analytic Rarefaction 1.3". In: *University of Georgia, Athens.*

Huttenhower, Curtis et al. (2012). "Structure, function and diversity of the healthy human microbiome". In: *Nature* 486.7402, pp. 207–214. ISSN: 0028-0836. DOI: 10.1038/nature11234. eprint: NIHMS150003. URL: http://www.nature.com/doifinder/10.1038/nature11234.

Kalinowski, Steven T (2005). "hp-rare 1.0: a computer program for performing rarefaction on measures of allelic richness". In: *Molecular Ecology Notes* 5.1, pp. 187–189.

Kruskal, William H and W Allen Wallis (1952). "Use of ranks in one-criterion variance analysis". In: *Journal of the American statistical Association* 47.260, pp. 583–621.

McMurdie, Paul J and Susan Holmes (2014). "Waste not, want not: why rarefying microbiome data is inadmissible". In: *PLoS Comput Biol* 10.4, e1003531.

Mulder, CPH et al. (2004). "Species evenness and productivity in experimental plant communities". In: *Oikos* 107.1, pp. 50–63.

Oksanen, Jari et al. (2016). *vegan: Community Ecology Package.* R package version 2.4-0. URL: https://CRAN.R-project.org/package=vegan.

Robinson, Mark D, Davis J McCarthy, and Gordon K Smyth (2010). "edgeR: a Bioconductor package for differential expression analysis of digital gene expression data". In: *Bioinformatics* 26.1, pp. 139–140.

Shannon, C. E. (1948). "A mathematical theory of communication". In: *The Bell System Technical Journal* 27.3, pp. 379–423. ISSN: 0005-8580. DOI: 10.1002/j.1538-7305.1948.tb01338.x.

Simpson, Edward H (1949). "Measurement of diversity." In: *Nature.*

Sunagawa, Shinichi et al. (2015). "Structure and function of the global ocean microbiome". In: *Science* 348.6237, p. 1261359.

Szpiech, Zachary A, Mattias Jakobsson, and Noah A Rosenberg (2008). "ADZE: a rarefaction approach for counting alleles private to combinations of populations". In: *Bioinformatics* 24.21, pp. 2498–2504.

Vavrek, Matthew J. (2011). "fossil: palaeoecological and palaeogeographical analysis tools". In: *Palaeontologia Electronica* 14.1. R package version 0.3.0, 1T.

Yatsunenko, Tanya et al. (2012). "Human gut microbiome viewed across age and geography". In: *Nature* 486.7402, pp. 222–227.
